# Differential Expression of Polyamine Pathways in Human Pancreatic Tumor Progression and Effects of Polyamine Blockade on Tumor Microenvironment

**DOI:** 10.3390/cancers13246391

**Published:** 2021-12-20

**Authors:** Sai Preethi Nakkina, Sarah B. Gitto, Veethika Pandey, Jignesh G. Parikh, Dirk Geerts, Hans Carlo Maurer, Kenneth P. Olive, Otto Phanstiel, Deborah A. Altomare

**Affiliations:** 1 Burnett School of Biomedical Sciences, College of Medicine, University of Central Florida, Orlando, FL 32827, USA; SaiPreethi.Nakkina@ucf.edu; 2 Ovarian Cancer Research Center, Division of Gynecology Oncology, Department of Obstetrics and Gynecology, Perelman School of Medicine, University of Pennsylvania, Philadelphia, PA 19104, USA; Sarah.Gitto@pennmedicine.upenn.edu (S.B.G.); Veethika.Pandey@pennmedicine.upenn.edu (V.P.); 3 Center for Cellular Immunotherapies, University of Pennsylvania, Philadelphia, PA 19104, USA; 4 Department of Pathology and Laboratory Medicine, Abramson Cancer Center, Perelman School of Medicine, University of Pennsylvania, Philadelphia, PA 19104, USA; 5 Department of Pathology, Orlando VA Medical Center, 13800 Veterans Way, Orlando, FL 32827, USA; jignesh.parikh@va.gov; 6 Department of Medical Biology, Academic Medical Center, University of Amsterdam, Meibergdreef 9, 1105 AZ Amsterdam, The Netherlands; Dirk@glycostem.com; 7 Internal Medicine II, School of Medicine, Technische Universität München, 81675 Munich, Germany; carlo.maurer@tum.de; 8 Department of Medicine, Vagelos College of Physicians and Surgeons, Columbia University Irving Medical Center, New York, NY 10032, USA; kenolive@columbia.edu; 9 Herbert Irving Comprehensive Cancer Center, Columbia University Irving Medical Center, New York, NY 10032, USA; 10 Department of Medical Education, College of Medicine, University of Central Florida, Orlando, FL 32826, USA

**Keywords:** pancreatic ductal adenocarcinoma, polyamine metabolism, tumor microenvironment, immune suppression, polyamine blockade therapy, survival, macrophage, CD86, DFMO, polyamine transport inhibitor

## Abstract

**Simple Summary:**

Pancreatic cancer has a five-year survival rate of less than 8% and is the fourth leading cause of cancer death in the United States. Existing therapeutics have failed to improve pancreatic ductal adenocarcinoma (PDAC) patient outcomes. There has been success with other tumor types in targeting aberrant polyamine upregulation as a therapeutic strategy. The present study identified dysregulation of polyamine pathways to be evident in human PDAC progression. Additionally, reduced survival of pancreatic cancer patients was associated with increased expression of specific polyamine-related genes. Polyamine blockade therapy significantly increased overall survival of pancreatic tumor-bearing mice, along with macrophage presence (F4/80) and significantly increased T-cell co-stimulatory marker (CD86) in the tumor microenvironment. Based on these findings, we hypothesized that a polyamine blockade therapy could potentially prime the tumor microenvironment to be more susceptible to existing therapeutics. Future studies which test polyamine blockade therapy with existing therapeutics could increase the molecular tools available to treat PDAC.

**Abstract:**

Pancreatic cancer is the fourth leading cause of cancer death. Existing therapies only moderately improve pancreatic ductal adenocarcinoma (PDAC) patient prognosis. The present study investigates the importance of the polyamine metabolism in the pancreatic tumor microenvironment. Relative mRNA expression analysis identified differential expression of polyamine biosynthesis, homeostasis, and transport mediators in both pancreatic epithelial and stromal cells from low-grade pancreatic intraepithelial neoplasia (PanIN-1) or primary PDAC patient samples. We found dysregulated mRNA levels that encode for proteins associated with the polyamine pathway of PDAC tumors compared to early lesions. Next, bioinformatic databases were used to assess expression of select genes involved in polyamine metabolism and their impact on patient survival. Higher expression of pro-polyamine genes was associated with poor patient prognosis, supporting the use of a polyamine blockade therapy (PBT) strategy for inhibiting pancreatic tumor progression. Moreover, PBT treatment of syngeneic mice injected intra-pancreatic with PAN 02 tumor cells resulted in increased survival and decreased tumor weights of PDAC-bearing mice. Histological assessment of PBT-treated tumors revealed macrophage presence and significantly increased expression of CD86, a T cell co-stimulatory marker. Collectively, therapies which target polyamine metabolism can be used to disrupt tumor progression, modulate tumor microenvironment, and extend overall survival.

## 1. Introduction

Pancreatic cancers have a low five-year survival rate of less than 8% [1]. By the year 2030, pancreatic cancer is projected to be the second leading cause of cancer related deaths in the US [2]. Since 1997, gemcitabine treatment in PDAC patients has significantly improved survival [3]. Newer treatments such as FOLFIRINOX and gemcitabine/nab-paclitaxel have shown a modest increase in patient survival, with no treatment increasing the median survival of metastatic PDAC patients by more than 12 months [4]. The failure of therapies targeting molecular pathways in PDAC has been in part attributed to the dense desmoplastic reaction, which is characteristic of PDAC, and the upregulation of alternate compensatory pathways [5]. 

Immune suppressive myeloid cells dominate the pancreatic tumor microenvironment, aiding in tumor cell immune evasion from cytotoxic T lymphocytes, suggesting that immunotherapies may be a promising approach in PDAC treatment [6]. However, immunotherapies alone have not consistently shown success in PDAC treatment to date [7,8]. A promising approach is combining immunotherapies with existing therapeutics to increase synergy. 

Prior work has shown that targeting polyamines in breast tumors resulted in immune modulation [9]. Therapies that improve the immune response in PDAC would be a significant advance. Polyamines are polycationic aliphatic amines, whose metabolism is upregulated in cancers with excessive metabolic demands [10]. The expression of the native polyamines (putrescine, spermidine, and spermine) is important for regulation of cellular processes including RNA processing, autophagy, metastasis, tumorigenesis, translation, maintenance of chromatin structure, and immune response [11,12,13,14,15]. Polyamine metabolism in healthy cells is a tightly regulated system, which balances biosynthesis, catabolism, and transport to maintain homeostasis [16]. A recent review from our group outlined the role of polyamine metabolism in PDAC and suggested that spermine may be involved in establishing immune privilege [16]. Localized polyamine depletion may initiate the simultaneous targeting of tumor-promoting pathways that rely upon polyamines while potentially modulating immune privilege.

The most widely studied polyamine biosynthesis inhibitor, difluoromethylornithine (DFMO), inhibits ornithine decarboxylase (ODC) activity. ODC catalyzes the rate limiting step of polyamine biosynthesis involving the conversion of ornithine to putrescine [17,18]. Tumor cells can escape the therapeutic effects of DFMO by importing polyamines from the extracellular environment [10,19,20]. Therefore, polyamine blockade therapy (PBT), which combines a polyamine synthesis inhibitor such as DFMO with a polyamine transport inhibitor (PTI), is required for efficient intracellular polyamine depletion [10,20,21]. Previously, our group showed that PBT can decrease intracellular polyamine levels and cell viability in pancreatic cancer cells [10,21]. This approach provides a way to limit polyamines in the tumor microenvironment and may provide an important adjuvant technology to current pancreatic cancer treatments. 

PBT-associated anti-tumor immune response has been tested in colon carcinoma and melanoma preclinical studies [15,20]. Studies have also shown the success of combining PBT with anti-PD-L1 therapy in mammary carcinoma and melanoma xenograft models [22]. Furthermore, preclinical studies from our group using PBT showed increased survival of pancreatic tumor-bearing mice [10]. However, questions remain regarding the immunomodulatory effects of polyamine blockade therapy in PDAC. 

Since the polyamine spermine is a known immune suppressant [23], we hypothesized that PDAC creates a spermine gradient (i.e., a polyamine shield) around the tumor to contribute to immune privilege. This would provide a mechanism to explain how PDAC tumors remain immunologically quiescent via their upregulated polyamine metabolism. This report shows aberrant expression of several mediators of polyamine pathways in specific stages of PDAC progression. This pro-polyamine expression pattern was shown to inversely correlate with patient prognosis. We also show for the first time an immune-regulatory effect of PBT in PDAC. Overall, results from this study provide evidence that supports targeting the PDAC tumor microenvironment with PBT, thereby disrupting the ‘polyamine shield’ and interfering with immune privilege to increase immune cell infiltration into the local PDAC tumor environment.

## 2. Materials and Methods

### 2.1. Materials

Synthesis of the Trimer44NMe PTI has been previously described [21]. DFMO was obtained as a gift from Patrick Woster at the Medical University of South Carolina. 

### 2.2. Cell Culture

Murine PAN 02 pancreatic tumor cells were obtained from the Division of Cancer Treatment and Diagnosis (DCTD) Tumor Repository (National Cancer Institute, Frederick, MD, USA). PAN 02 pancreatic cancer cells were routinely screened by PCR for mycoplasma (eMycoTM, iNtRON Biotechnology, Seongnam, Korea). Cells were cultured in DMEM media (Corning, NY, USA; MT15013CV) containing 10% fetal bovine serum (FBS) and 1× Penicillin-Streptomycin, and incubated in a 5% CO_2_ incubator at 37 °C.

### 2.3. mRNA Study

Human primary PDAC and low-grade PanIN samples were collected from a total of 223 patients with PDAC who underwent surgery at the Columbia Pancreas Center. Frozen pancreas tissue banked at the Columbia University Medical Center were used. Only samples with a PDAC diagnosis for which intact RNA was available were selected. The diagnosis of all samples was confirmed by an independent gastro-intestinal pathologist prior to microdissection. For each tissue sample analyzed, epithelial and stromal cells were micro-dissected and isolated, yielding matched pairs for each patient. Whole transcriptome RNA amplification using the NuGEN Ovation RNA-Seq System V2 kit was performed on total RNA and yielded several µg of cDNA. The cDNAs were sequenced on an Illumina HiSeq 3500 to a depth of 30 million 100 bp single-end reads. In total, 197 epithelial with 100 matching stromal samples from primary PDACs and 26 epithelial with 23 matching stromal samples from low-grade PanIN were used in this study. Using GraphPad Prism, the data were analyzed using box and whisker plots to represent the medium, the interquartile, and the total range. Statistical significance between means was established via a two-way ANOVA with Tukey’s multiple comparison test (*p* < 0.05 [*], *p* < 0.01 [**], *p* < 0.001 [***]).

### 2.4. Transcriptome Analyses

Genome-wide mRNA expression profiles of human pancreatic cancer datasets were from the public Gene Expression Omnibus (GEO) dataset at the NCBI website (http://www.ncbi.nlm.nih.gov/geo/, accessed on 3 November 2021). We found 6 mixed datasets containing cancerous and matching normal pancreas samples: Badea-78 (GSE15471), Hussain-130 (GSE62452), Topal-131 (GSE62165), Wang-51 (GSE16515), Wu-32 (GSE32676), and Zhang-90 (GSE28735), all from Affymetrix platforms, and were downloaded and normalized using rma or MAS5.0 as described previously [24]. All analyses were performed using R2, a genomics analysis and visualization platform (http://r2.amc.nl, accessed on 3 November 2021). The R2 TranscriptView genomic analysis and visualization tool (http://r2.amc.nl) was used to select probe-sets. Probes had to show unique mapping in an anti-sense position within late coding exons and/or the 3′ UTR of the gene. When multiple correct probe-sets were available for a gene, the probe-set with the highest average expression and the highest number of present calls for that dataset was used. The selected SMS probe-set met these criteria and in no cases did additional probe-sets show conflicting results for that dataset. SMS mRNA expression differences between pancreas cancer and normal samples in the 6 mixed datasets was determined using the non-parametric Kruskal–Wallis test. Results were considered statistically significant when *p* < 0.05.

Gene expression profiles and survival characteristics of select genes, or those involved in either polyamine synthesis, homeostasis, or transport, were sourced from The Cancer Genome Atlas Pancreatic Adenocarcinoma (TCGA-PAAD) dataset downloaded using the University of California Santa Cruz (UCSC) Xena browser (https://xena.ucsc.edu/, accessed on 3 November 2021). All samples were primary PDAC (*n* = 178), and expression was normalized to log-transformed transcripts per million pseudocounts (log2(TPM + 1)). Patient samples were segregated into tertiles based on low (*n* = 59), medium (*n* = 59), or high expression (*n* = 60) for each gene. Overall survival was then plotted as a Kaplan–Meier curve using the survminer (v0.4.9) R package. Significance of survival differences were calculated using a log-rank test, and the hazard ratio was calculated by univariate Cox regression. Genes whose change in expression was associated with an impact on survival were represented. Results were considered statistically significant when *p* < 0.05 (*p* < 0.05 [*], *p* < 0.01 [**], *p* < 0.001 [***]).

### 2.5. In Vivo Studies

In vivo experiments were performed in accordance with the Guide for the Care and Use of Laboratory Animals, with approval of the University of Central Florida Institutional Animal Use and Care Committee (protocol PROTO202000011). To test DFMO + PTI in vivo, 0.5 × 10^6^ PAN 02 murine pancreatic cancer cells were orthotopically injected into the pancreas of immune-competent C57Bl/6 mouse (6–8 weeks old) obtained from Jackson Laboratories, ME. One to two weeks after the surgery, mice were randomized into treatment groups. DFMO was dosed in the drinking water (either 0.25% or 1% *w/v* DFMO) and the PTI (either 1.8 mg/kg or 4 mg/kg) was injected intraperitoneally for 5 days followed by two days off each week of the treatment.

In the survival study, one week after tumor seeding, male and female mice (*n* = 4–5 of each sex, 9–10 animals per treatment group) received a treatment regimen until the mouse succumbed to disease or until euthanasia due to a combined poor health score (such as >20% body weight, hunched appearance, and/or lethargy). Sample size estimation for survival experiment power analysis was conducted using data from our previous studies. Hazard ratio of each treatment vs. the control group was estimated by using Cox regression. Log-rank test was used to estimate the sample size for each group to detect hazard ratios with a power of 85% and a significance level of α = 0.05. Significance of survival differences was calculated using a log-rank test (*p* < 0.01 [**], *p* < 0.001 [***]). Median survival of tumor-bearing mice in each treatment group was also calculated. 

Tumor phenotype across all treatment groups was evaluated in a fixed termination (fixed term) study. Since the survival study showed no sex-based differences, 8–9 female mice were used per group, and each treatment was administered for 3 weeks. Animals were necropsied, tumors were weighed, and histological comparisons of the primary pancreatic tumor were conducted using hematoxylin and eosin staining. Because in situ tumors were limited in size for the combination treatment of that fixed term study, a separate fixed term study used 5 female mice per group, and 2 weeks after tumor seeding, each treatment was administered for 2 weeks. At study termination, tumor weights were recorded, followed by histological comparisons of the primary pancreatic tumor using immunohistochemical (IHC) staining. Results are reported as mean ± SD. To compare each mean to the control mean, a one-way ANOVA with post hoc Dunnett’s multiple comparison was used to analyze statistical significance between tumor weights (*p* < 0.05 [*], *p* < 0.01 [**], *p* < 0.001 [***]).

### 2.6. Histological Analysis

Tissues were fixed in 10% neutral buffered formalin (Surgipath Leica, Buffalo Grove, IL, USA), embedded in paraffin, sliced in 5 μm sections, and dried at 65 °C for 1 h. Slides were used for IHC staining using Polymer Refine Detection reagents (Leica) on a Bond-Max immunostainer (Leica, Buffalo Grove, IL, USA). Antigen retrieval for IHC was optimized with sodium citrate (pH 6.0) or EDTA (pH 9.0). Primary antibodies included F4/80 (Cell Signaling Technology, Danvers, MA, USA; 70076S), CD86 (Cell Signaling Technology, Danvers, MA, USA; 19589S) and Ym1 (STEMCELL Technologies, Vancouver, Canada; 60130). Stained sections were assessed by a pathologist and poorly differentiated tumor regions were chosen for quantification. IHC staining was quantified using the Keyence BZ-X800 analysis software. Where possible, serial sections were imaged, and samples from three mice per treatment group and two fields of view per histology specimen were used for quantification. Results are reported as mean ± SD. To compare each mean with every other mean, a one-way ANOVA with Tukey’s multiple comparison was used to analyze statistical significance between means (*p* < 0.05 [*], *p* < 0.01 [**], *p* < 0.001 [***]).

## 3. Results

### 3.1. mRNA Expression Depicts Altered Polyamine Metabolism in PDAC versus Pre-Cursor PanIN Lesions

**Figure 1:**
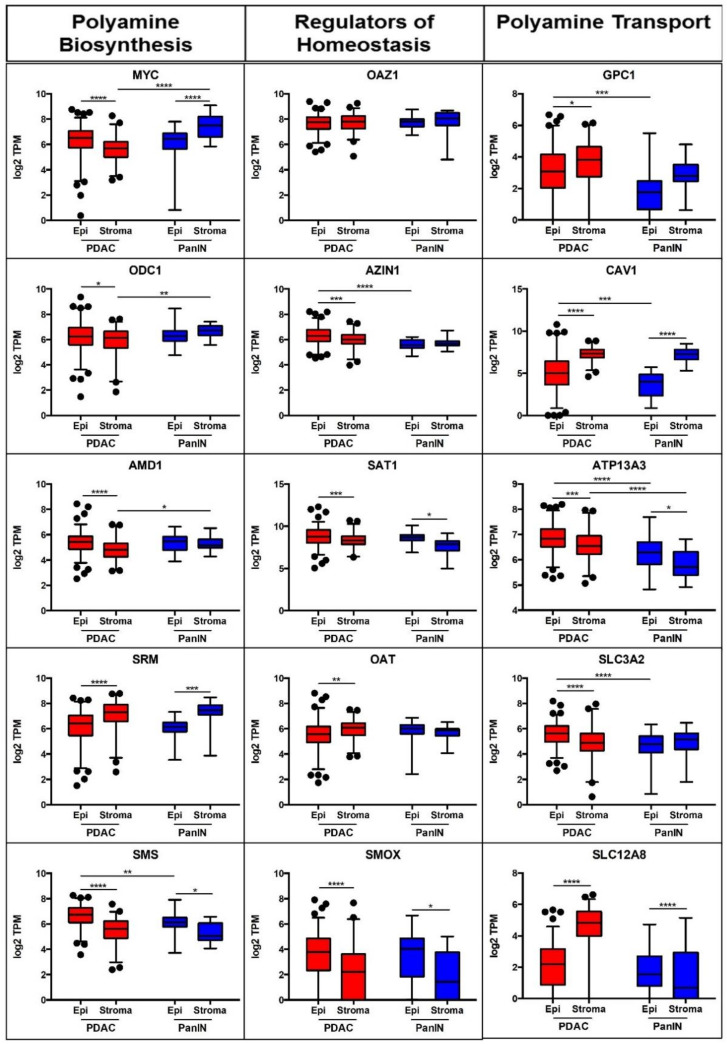
Relative mRNA expression of select polyamine biosynthesis, homeostasis, and transport genes altered in PDAC. Expression of each indicated gene in the epithelial (Epi) and stroma compartments isolated from human PanIN-1 or PDAC samples by laser capture microdissection are represented as log2 scale of transcripts per million (TPM). Approximately 1000 cells per sample were captured and analyzed per materials and methods descriptions. *p* value: * < 0.05, ** < 0.01, *** < 0.001, **** < 0.0001.

**Figure 2:**
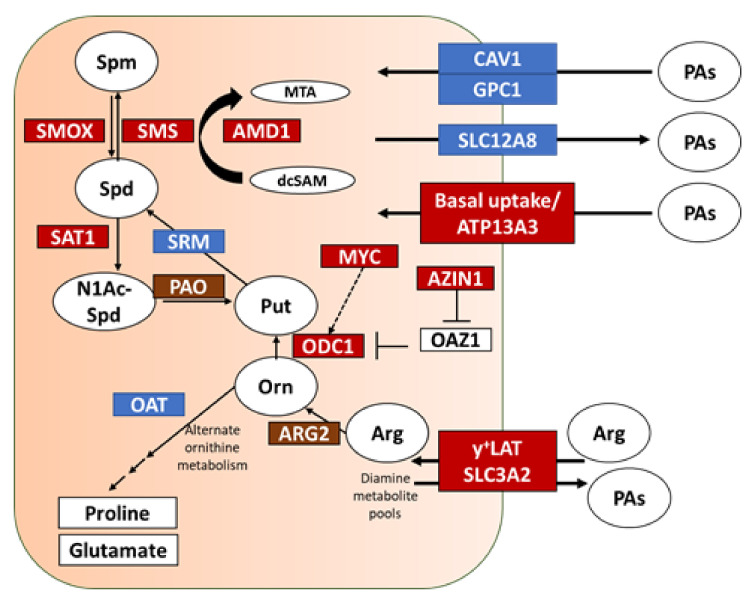
Polyamine dysregulation model in PDAC. Graphical summary of key players in polyamine regulation within a cell, highlighting upregulated (red) and downregulated (blue) gene expression products in PDAC epithelium versus stroma. Abbreviations: Arg: Arginine; ARG2: Arginase 2; ATP13A3: an ATPase involved in polyamine transport; AZIN1: Antizyme Inhibitor 1; CAV1: Caveolin 1; GPC1: Glypican 1; Orn: Ornithine; OAZ1: Antizyme 1; ODC1: Ornithine Decarboxylase 1; Put: Putrescine; SLC3A2: Solute transporter 3A2 (subunit of the diamine exporter DAX); SMS: Spermine Synthase; Spd: Spermidine; Spm: Spermine; SRM: Spermidine Synthase; y + LAT: cationic amino acid transporter, subunit of the diamine exporter DAX; OAT: Ornithine Aminotransferase; PAO: Polyamine Oxidase; SMOX: Spermine Oxidase; MYC: Myc Proto-Oncogene Protein; SAT1: Spermidine/Spermine N1-Acetyltransferase 1; AMD1: Adenosylmethionine Decarboxylase 1; MTA: 5′-methylthioadenosine; dcSAM: decarboxylated S-adenosylmethionine; SLC12A8: solute carrier family 12 member 8; N1Ac-Spd: N1-Acetylspermidine; PA: polyamine/polyamine metabolites.

Polyamine metabolism in healthy cells is a tightly regulated system, which balances biosynthesis, catabolism, and transport to maintain homeostasis [16]. To better understand polyamine metabolism in human PDAC tumors, we determined the relative mRNA expression patterns of known genes involved in polyamine metabolism and transport as well as other genes of interest. Laser tissue microdissection of the tumor compartments (epithelium and stroma) were compared over tumor progression (PanIN versus PDAC) in clinical PDAC patient samples. Here, PanIN-1 samples were used as baseline control instead of normal pancreas which contain ~90% acinar cells and have most of their mRNA transcripts dedicated for digestive enzyme production, making them a poor comparison. Data revealed aberrant polyamine dysregulation during PDAC progression. The data for each mRNA are shown in Figure 1 and the affected pathway illustrated in the polyamine metabolism model in Figure 2.

First, we assessed mediators of polyamine biosynthesis. *MYC* is a known oncogene and regulator of polyamine synthesis [25]. MYC is a transcriptional activator of ODC1 which facilitates the synthesis of the diamine putrescine. AMD1 produces decarboxylated S-adenosylmethionine (dcSAM), which is a building block used in the synthesis of the higher order polyamines: spermidine and spermine. SRM and SMS are biosynthetic enzymes which catalyze the conversion of putrescine and spermidine into spermidine and spermine, respectively [26,27,28,29,30]. Of the five genes involved in polyamine synthesis described here, MYC, ODC1, AMD1, and SMS show increased expression in the epithelial compartment of PDAC samples (Figure 1). These data indicate an increase in overall polyamine synthesis gene signature in the epithelial tumor compartment of PDAC, in comparison to stromal compartment of PDAC. 

Next, we assessed regulators of polyamine homeostasis. Antizyme (OAZ1) targets ODC1 for proteasomal degradation. Spermine is toxic to cells at high concentration and its cellular level is tightly controlled via catabolism [26,27]. Upon increase in intracellular polyamine levels, SMOX and SAT1 both play a role in conversion of higher order polyamines such as spermine and spermidine to a lower order [31]. OAT is involved in alternate ornithine metabolism, shunting ornithine away from polyamine biosynthesis [32]. AZIN1 limits the polyamine synthesis inhibition executed by OAZ1 [26,27]. Our data show a reduction in polyamine synthesis repression (AZIN1), an increase in enzymes that break down higher order polyamines (SMOX, SAT1), and a decrease in alternate polyamine metabolism (OAT) in the epithelial compartment of PDAC. Collectively, these studies revealed a ‘pro-polyamine’ gene signature in PDAC.

Polyamine transport is another mode of regulating intracellular polyamine levels. Glypican-1 (GPC1) is the anchoring protein associated with heparan sulfate proteoglycan (HSPG) mediated polyamine import [33,34]. Caveolin 1 (CAV1) is a negative regulator of polyamine import [35,36]. We recently showed ATP13A3 to be involved in polyamine import in PDAC [19,37]. SLC3A2 is known to export polyamines from human cells [38]. SLC12A8 has been suggested as an ornithine/polyamine transporter [39,40,41] and more recently as a nicotinamide mononucleotide transporter [42]. The epithelial (tumor) portion of the PDAC samples showed an increase in the expression of ATP13A3 (polyamine import) and SLC3A2 (diamine exporter) and lower expression of CAV1. This pattern of polyamine transport associated genes suggested increased transport activity in PDAC tumors. The stromal compartment of PDAC showed higher expression of GPC1 and SLC12A8 which support polyamine import. The data indicate the presence of specific polyamine transport regulation in the different compartments of PDAC and support the reliance of pancreatic cancer cells on ATP13A3-mediated polyamine import, as we previously reported [19].

Overall, a trend of increased expression of genes involved in polyamine synthesis, dysregulated polyamine homeostasis, and import in epithelial compartment of PDAC in comparison to stroma is observed. SMS, AZIN1, GPC1, CAV1, ATP13A3, and SLC3A2 show differences in expression between PanIN and PDAC in the epithelial compartment while MYC, ODC1, AMD1, and ATP13A3 showed differences specific to the stromal compartment. These data are indicative of changes in polyamine metabolism during PDAC progression from early PanIN stage and support our hypothesis that aberrant polyamine metabolism is an important target in PDAC therapy.

### 3.2. Prognosis Correlates with Expression of Select Polyamine-Related Genes in Pancreatic Tumors

Upregulation of SMS expression in PDAC, as seen in Figure 1, is suggestive of increased spermine supply in PDAC and potential increase in spermine associated immune suppression via spermine export. To test this hypothesis, we looked at relative SMS expression in six datasets with cancerous pancreas and matched normal pancreas samples. In all six datasets, SMS was significantly higher in tumor cells than in normal pancreas, in complete agreement with our own microdissection results (Supplemental Figure S1).

**Figure 3:**
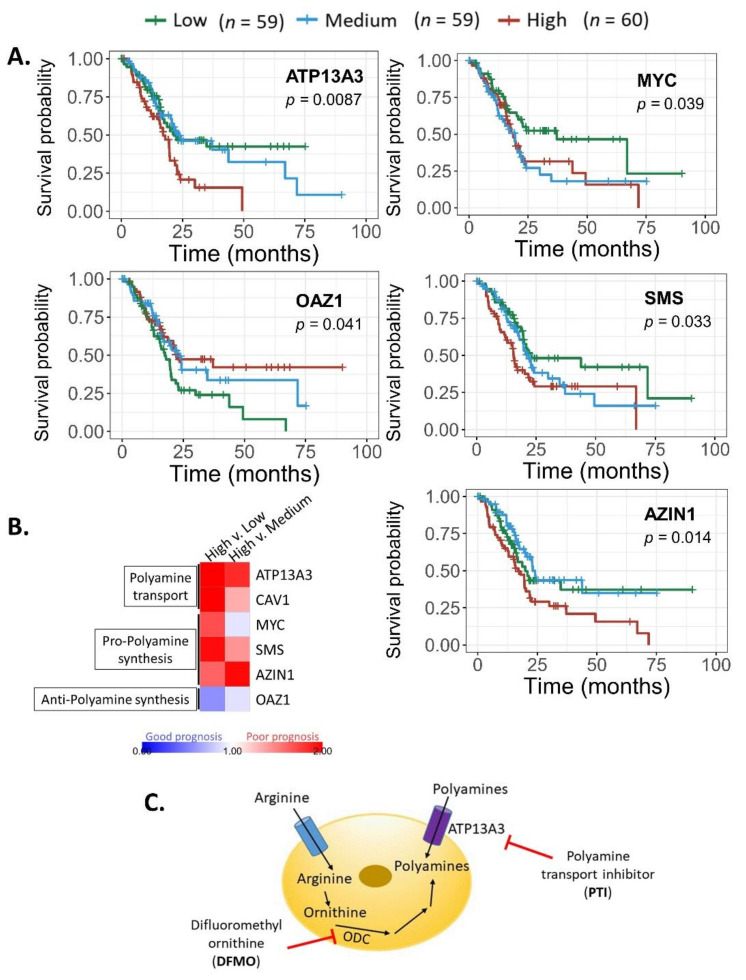
Select polyamine gene expression in human PDAC tumors were associated with patient survival and prognosis. MYC, SMS, AZIN1, OAZ1, and ATP13A3 expression levels were correlated to patient outcomes in the TCGA dataset. (**A**) Survival of patients in the upper, median, and lower tertiles of gene expression was plotted in Kaplan–Meier curves. (**B**) Hazard ratio associated with high (upper tertile) gene expression versus either low (lower tertile) or medium (median tertile) gene expression was quantified as a ratio and depicted in a heatmap. (**C**) Schematic of polyamine blockade therapy strategy showing the nodes of DFMO and PTI intervention, which inhibit polyamine synthesis and import, respectively.

We next analyzed whether the mRNA expression of polyamine pathway components correlated to clinical outcomes. Figure 3A represents survival curves of polyamine genes whose expression differences resulted in significant survival differences. As expected, high expression of MYC, SMS, AZIN1 (pro-polyamine synthesis), and ATP13A3 (polyamine transport) are associated with poorer survival. In contrast, high expression of OAZ1 (anti-polyamine synthesis) was associated with improved patient survival. Hazard ratio (linked to poor prognosis) associated with low, medium, and high expression of individual genes was assessed. The heat map in Figure 3B compares the hazard ratio of high gene expression to low or medium expression. Figure 3B depicts a trend of poorer patient prognosis with higher expression of MYC, SMS, AZIN1, and ATP13A3, and improved prognosis with OAZ1 expression as expected. 

The bioinformatic data from the TCGA dataset provide a robust extension of our result, and confirm that SMS expression is beneficial to PDAC tumor growth and progression. More importantly, this pattern (high SMS, high MYC, high AZIN1, high ATP13A3, low OAZ1) was significantly predictive of poor clinical outcome and prognosis (Figure 3A,B).

### 3.3. Polyamine Blockade Therapy Improves Pancreatic Cancer Outcome In Vivo

Data from the present investigation (Figure 1 and Figure 3A,B) and previous work from our laboratory led us to further test polyamine blockade therapy in vivo [10]. Since both the increased expression of polyamine synthesis and transport genes are linked to poor prognosis, we targeted both modalities of polyamine availability using both DFMO and PTI (Figure 3C).

For in vivo testing, we identified a murine pancreatic cancer cell line PAN 02 with high polyamine transport activity and sensitivity to DFMO [19]. The polyamine transport inhibitor (PTI) Trimer44NMe competitively inhibited import of ^3^H-labeled spermidine into PDAC cells and works synergistically with DFMO to deplete PDAC cells of intracellular polyamine pools [19].

**Figure 4:**
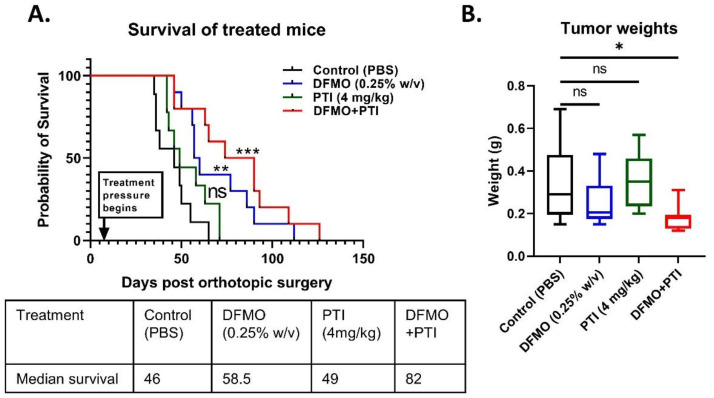
Survival was increased and tumor weights were decreased in PBT treated PAN 02 tumor-bearing mice. (**A**) Kaplan–Meier curves and associated median survival of mice treated as indicted (*n* > 9 mice per group). DFMO treatment alone showed a significant improvement in survival compared to control (Log-Rank test, *p* = 0.0029), although PBT showed the greatest improvement in survival (Log-Rank test, *p* = 0.001) among the treatment groups. (**B**) Fixed termination timepoint study showing tumor weights from treated mice (*n* > 8 mice per group). *p* value: * < 0.05, ** < 0.01, *** < 0.001.

To test polyamine blockade therapy in vivo, syngeneic C57Bl/6J mice with orthotopically injected PAN 02 tumor cells and were treated with either control (PBS), DFMO (0.25% *w/v*), PTI (4 mg/kg), or a combination of DFMO and PTI (PBT) one week after tumor cell seeding. The current study tested Trimer44NMe at a higher dose than in previous PDAC models, and animals were treated continuously, as described in the methods section. PTI alone did not show any remarkable difference in survival compared to control group (Figure 4A). DFMO alone showed a significant improvement in survival compared to control (log-rank test, *p* = 0.0029), although PBT showed the greatest improvement in survival (log-rank test, *p* = 0.001) among the treatment groups. PBT treatment increased the median survival to 82 weeks from 46 weeks for the control group, whereas DFMO alone had a modest improvement (58.5 weeks).

In conjunction with the survival study, a fixed-term study of tumor-bearing mice was conducted using DFMO (0.25%) and PTI (4 mg/kg) for three weeks of treatment. After three weeks from the beginning of the treatments, tumors were excised, and weights were recorded. PBT showed a significant decrease in tumor weight when compared to control treated mice (*p* = 0.035), in contrast to DFMO (*p* = 0.3697) and PTI (*p* = 0.9614) single agent treatments. Overall, PBT showed remarkable anti-tumor effects in vivo in the described orthotopic PDAC model. 

### 3.4. PBT Increases the Expression of T Cell Co-Stimulatory Marker CD86 in the PDAC Tumor Microenvironment

**Figure 5:**
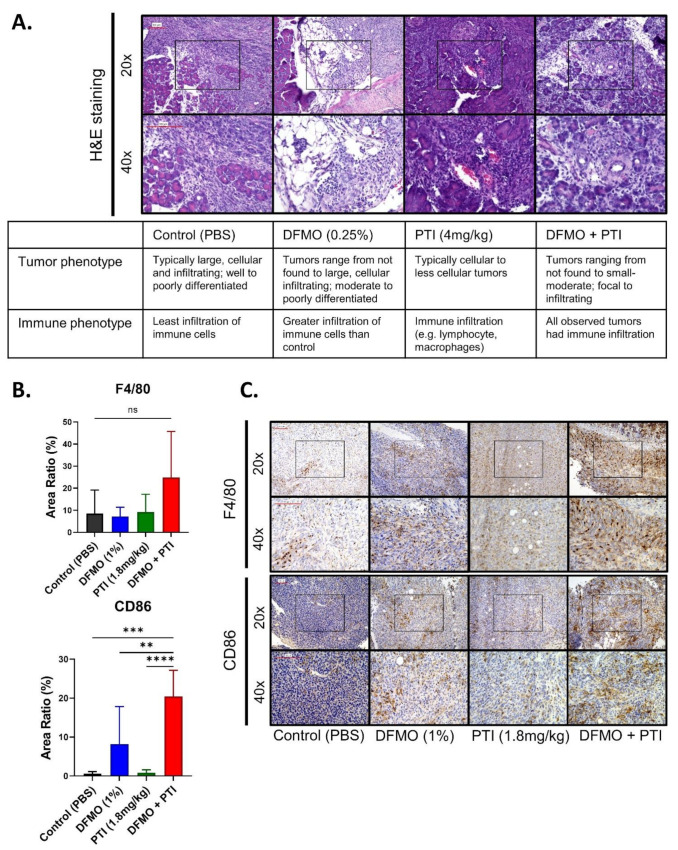
Pathological assessment shows differences in tumor phenotype and presence of infiltrating immune cells in the mice treated with DFMO and/or PTI (Trimer44NMe). (**A**) Representative Hemotoxylin and Eosin (H&E) stained sections assessed for tumor phenotype and microenvironment with respect to 0.25% (*w/v*) DFMO and/or 4 mg/Kg PTI (Trimer44NMe) treatments. (**B**) Quantification of expression of F4/80 and CD86 across 1% (*w/v*) DFMO and/or 1.8 mg/kg PTI (Trimer44NMe) treatment groups. (**C**) Representative immunohistochemistry images of F4/80 and CD86 stained 1% (*w/v*) DFMO and/or 1.8 mg/kg PTI (Trimer44NMe)-treated pancreatic tumor sections imaged at 20× and 40× magnification. The 20× images contain inlays representing area captured at 40× magnification. Scale bars correspond to 100 μm. *p* value: ** < 0.01, *** < 0.001, **** < 0.0001.

Tumor sections from treated mice were stained with hematoxylin and eosin (H&E) and assessed for pathology (J.G.P.). Pathological assessment revealed that the tumor size was consistent with the tumor weights (Figure 4B), and the presence of immune infiltrate in PBT treated tumors (Figure 5A). The results prompted us to investigate specific immune-associated changes in larger tumor samples from a fixed term study using control, DFMO (1% *w/v*), PTI (1.8 mg/kg), and PBT (1% DFMO and 1.8 mg/kg PTI) [10].

Macrophages populate the PDAC environment and can facilitate tumor growth or hinder tumor progression [43,44]. Using immunohistochemistry, we observed a 2.9-fold increase in macrophage infiltration (F4/80 marker) in the DFMO + PTI (PBT) treatment group compared to all other treatment groups, although not a significant increase (*p* = 0.1475) (Figure 5B,C).

CD86 receptor expression was also evaluated due to its ability to stimulate naïve T cell activation by interaction of CD86-CD80 ligands with CD28 costimulatory molecules, and its common presence on antigen-presenting cells, including M1 and M2b macrophages. [45,46]. Increased expression of CD86 could indicate a greater propensity to stimulate an anti-tumor T cell response. Importantly, DFMO and DFMO + PTI treatment groups showed a 34.04-fold and 24.52-fold increase in CD86 expression compared to control and PTI treatment groups, respectively (Figure 5B,C). In contrast, M2 marker Ym1 did not exhibit significant expression changes in the tumor microenvironment with respect to the treatments (Supplemental Figure S1B,C), indicating that other antigen presenting cells and/or M1 macrophages were present in the tumors treated with DFMO, and even more prevalent with DFMO + PTI. Collectively, this analysis confirmed increased numbers of CD86+ antigen presenting cells and presence of macrophages, particularly in DFMO + PTI treated tumor-bearing mice, which was consistent with pathological assessments. Overall, these findings show that the efficacy of PBT in PDAC could be in part through modulation of the tumor-immune cell microenvironment.

## 4. Discussion

PDAC gains many survival advantages via polyamine dysregulation. We show that epithelial cells in both PanIN-1 and PDAC favor spermine production (Figure 1). In cells heavily engaged in spermine production high SMOX expression is needed to help maintain spermine homeostasis. Indeed, the PDAC epithelial samples showed this high SMS and high SMOX mRNA expression pattern, suggesting increased spermine production by these cells.

If PDAC cells rely on polyamines to support their growth and immune privilege, then high expression of proteins involved in polyamine transport would be expected. Higher expression of GPC1 and ATP13A3 (both associated with polyamine transport) were found in PDAC epithelia than in PanIN-1 epithelia. In contrast, lower expression of Cav1 (a negative regulator of polyamine import) was observed in both the PanIN-1 and PDAC epithelia compared to their respective stroma and was consistent with increased expression of genes regulating polyamine import in these regions. ATP13A3 expression was also higher in PDAC epithelia than PanIN epithelia, consistent with increased polyamine transport in PDAC relative to PanIN-1.

Bioinformatic data (Figure 3A,B; Supplemental Figure S1) from publicly available datasets showed that SMS expression is beneficial to PDAC tumor survival and progression, consistent with the mRNA pattern presented in Figure 1. Overall, aberrant expression of certain polyamine-related genes was shown to be associated with poorer survival of pancreatic cancer patients. Since polyamines in the PDAC microenvironment can facilitate dysregulated proliferation of tumor cells and possibly support the formation of an immunosuppressive environment, we tested how a PBT strategy would translate to anti-tumor success in PDAC.

This investigation revealed that 4 mg/kg of the PTI (Trimer44NMe) was tolerated in pancreatic tumor-bearing C57Bl/6J mice for over 100 days. PBT had increased anti-tumor efficacy over that of single agent DFMO or PTI alone in terms of increased survival and decreased tumor weight (Figure 4). These findings support further assessment of PBT strategies in preclinical and/or genetic models of PDAC tumor progression.

Since PBT treatment reduces intracellular polyamine levels, it is predicted to lower the polyamines available in the tumor microenvironment [21]. Decreased polyamine pools are postulated to then translate into an effect on the tumor microenvironment immune cell response. In the PDAC model, trending increase in macrophage infiltration and significantly increased CD86 expression (Figure 5B,C) observed are consistent with this rationale. These results are exciting, because they suggest that inhibiting polyamine biosynthesis and transport through a PBT strategy in PDAC can significantly increase survival by affecting the immune response. As CD86 is implicated in T-cell stimulation [45,46], PBT efficacy in PDAC may be T-cell dependent [15,20]. Other cancers have shown this T cell mediated response to PBT, but further work is needed to delineate this mechanism in PDAC [20]. 

Overall, while reports in the literature describe a role for polyamines in PDAC growth, the microenvironment, desmoplasia, and the immune response, no comprehensive model has been proposed that integrates these observations into a cohesive PDAC polyamine model [16]. Here, we proposed and tested a model where PDAC tumors could control their microenvironment via polyamine dysregulation. Importantly, our model provides a connection between cancer metabolic commitments and immune evasion and provides clearly defined targets for drug development (ODC, CAV1-mediated endocytosis, ATP13A3). Future work will apply these polyamine-targeted therapies to other human diseases, which rely on polyamines for intercellular communication [47,48].

## 5. Conclusions

Overall, the findings here provide evidence that other agents should be evaluated in combination with polyamine blockade therapy to improve its effectiveness as a cancer therapeutic. Among the possible targets that could be tested in future studies are additional immunomodulatory therapeutics. A success here would increase the diversity of molecular tools that could be used increase pancreatic cancer patient survival.

## Data Availability

A subset of the data included in the manuscript is publicly available at: https://www.ncbi.nlm.nih.gov/geo/query/acc.cgi?acc=GSE93326, accessed on 3 November 2021.

## Supplementary Materials

The following are available online at https://www.mdpi.com/article/10.3390/cancers13246391/s1, Figure S1: Spermine synthase expression in clinical pancreatic cancer samples and effects of polyamine blockade therapy on M2 macrophages in murine pancreatic cancer.
