# Supplementary material for: Differential Expression of Polyamine Pathways in Human Pancreatic Tumor Progression and Effects of Polyamine Blockade on Tumor Microenvironment
Source: Cancers (Basel). 2021 Dec 20;13(24):6391. doi: 10.3390/cancers13246391 (PMC8699198; doi:10.3390/cancers13246391)
Supplement: Supplementary file 1 [file cancers-13-06391-s001.zip › cancers-1479591-supplementary.pdf]

Differential Expression of Polyamine Pathways in Human Pancreatic Tumor Progression and Effects of Polyamine Blockade on Tumor Microenvironment

Sai Preethi Nakkina [1], Sarah B. Gitto [2,3,4], , Veethika Pandey [2,3,4], Jignesh G. Parikh [5], Dirk Geerts [6], Hans Carlo Maurer [7], Kenneth P. Olive [8,9], Otto Phanstiel IV [10,*] and Deborah A. Altomare [1,*]

A.

Dataset	Samples		SMS Expression	
	Tumor	Normal	Tumor / Normal	*P* Value
Badea-78	39	39	2.19	$8.9 \cdot 10^{-9}$
Hussain-130	69	61	1.43	$5.0 \cdot 10^{-4}$
Topal-131	118	13	9.81	$4.5 \cdot 10^{-9}$
Wang-51	36	15	2.51	$3.3 \cdot 10^{-5}$
Wu-32	25	6	2.74	$2.4 \cdot 10^{-3}$
Zhang-90	45	45	1.69	$2.1 \cdot 10^{-5}$

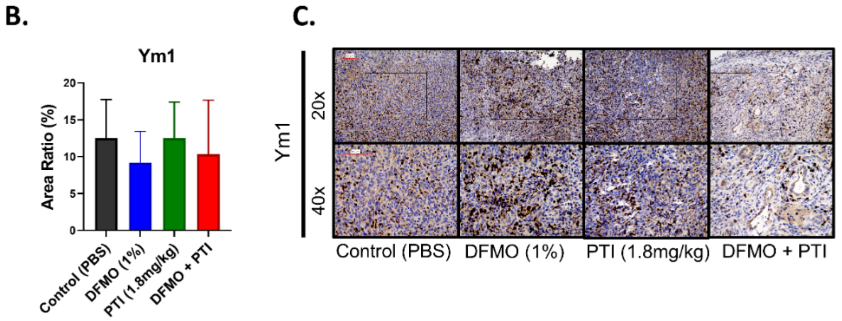

Supplemental figure S1. Spermine synthase expression in clinical pancreatic cancer samples and effects of polyamine blockade therapy on M2 macrophages in murine pancreatic cancer. (**A**) SMS mRNA expression in cancerous and normal pancreas tissue from six public mixed datasets. Kruskal-Wallis test was used for this comparison. (**B**) Quantification of Ym1 expression by IHC across treatment groups. (**C**) Immunohistochemistry images of Ym1 stained PBT-treated pancreatic tumor sections imaged at 20× and 40× magnification. 20× images contain inlays representing area captured at 40× magnification. Scale bars correspond to 100 μm.
